# Pediatric pancreatic teratoma: A case report and literature review

**DOI:** 10.1097/MD.0000000000018001

**Published:** 2019-11-15

**Authors:** Jian Wang, Yuan Yin, Zhaolun Cai, Chaoyong Shen, Xiaonan Yin, Xin Chen, Zhou Zhao, Bo Zhang

**Affiliations:** Department of Gastrointestinal Surgery, West China Hospital, Sichuan University, Chengdu 610041, Sichuan, China.

**Keywords:** pancreaticoduodenectomy, pediatric pancreatic teratoma, surgical resection

## Abstract

**Rationale:**

Pediatric pancreatic teratoma (PPT) is a rare tumor with unclear clinicopathologic features and treatment strategy.

**Patient concerns:**

A 13-month-old boy was admitted to the hospital with a complaint of a palpable epigastric mass.

**Diagnoses:**

The lesion was diagnosed as benign mature cyst teratoma via postoperative pathological examination.

**Interventions:**

Pylorus-preserving pancreaticoduodenectomy (PPPD) was performed on the patient. The entire mass was resected from the head of the pancreas and sent to the laboratory for frozen section evaluation.

**Outcomes:**

The patient was followed up for 15 months. He did not undergo recurrence or PPPD-related complications.

**Lessons:**

The differential diagnosis of retroperitoneal occupying lesions among children must consider mature cystic teratomas. Compete surgical resection combined with subsequent postoperative outpatient follow-up remains the primary choice for the management of PPTs.

PPTs are extremely rare tumors with unclear clinicopathologic features and treatment strategy. This study aims to explore the clinical characteristics of and treatment strategy for these tumors. We reported a 13-month-old patient with pancreatic teratoma who underwent pylorus-preserving PPPD. The operation lasted approximately 6 hours. The mass was completely removed, and the patient recovered uneventfully. Complete surgical resection combined with outpatient follow-up is the primary choice for the management of PPTs.

## 1 Introduction

Teratomas are common germ cell tumors among the pediatric population and are composed of tissues originating from all 3 dermal layers: endoderm, mesoderm, and ectoderm.^[1]^ The most common sites of these tumors are the sacrococcygeal region, ovaries, and mediastinum. However, teratomas derived from the pancreas are exceedingly rare among the pediatric population, resulting in difficulty in their preoperative diagnosis and treatment.^[2]^ Herein, we present the case of a pediatric patient with mature teratoma of the pancreatic head and who underwent pancreaticoduodenectomy (PD). We explore the safety and efficacy of PD for pediatric patients with teratomas in the pancreas to further understand this rare tumor.

This study was conducted in accordance with the principles outlined in the Declaration of Helsinki and authorized by the Ethics Committee of the hospital. Written consent was obtained from the parents before operation.

## 2 Case presentation

**Figure 1:**
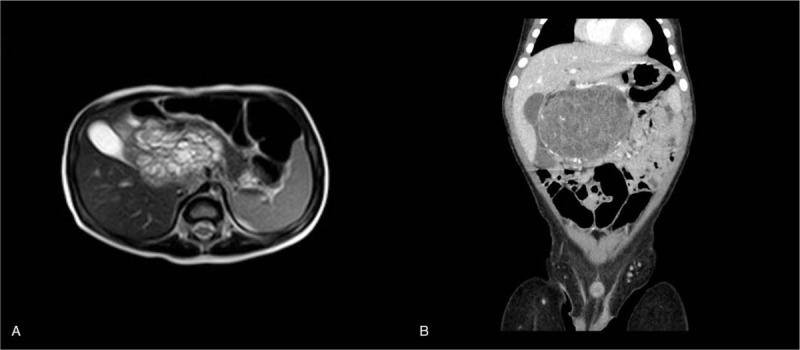
(A, B) Preoperative contrast-enhanced CT scan of the pediatric pancreatic teratoma (A B). The cystic solid mass in the right retroperitoneal region of the mid-upper abdomen is mainly cystic, with honeycomb septation and patchy shadow. The mass is closely related to the head of the pancreas. CT = computed tomography.

**Figure 2:**
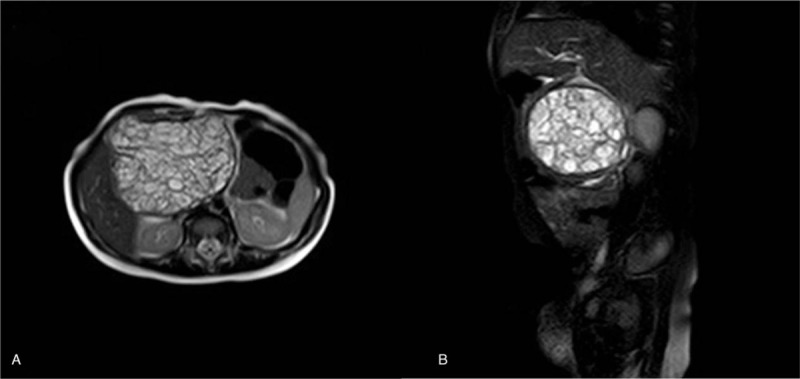
(A, B). Preoperative MRI scan of the pediatric pancreatic teratoma. Tumors contain a large amount of fat and sebum, and the outer membrane is intact. MRI = magnetic resonance imaging.

A 13-month-old boy was admitted to the institute due to a palpable abdominal mass that was incidentally noted by his mother during child health service. His physical examination was unremarkable, with only a round, hard, elastic, and rigid mass in his upper abdomen without tenderness. The parents did not complain of any discomfort or special family history. A solid cystic mass was revealed by abdominal contrast-enhanced computed tomography (CT) and magnetic resonance (MR) scans at the right side of the mid-upper retroperitoneum (Figs. 1 and 2). The tumor was approximately 9 cm × 6 cm × 5 cm in size and closely related to the pancreatic head. Laboratory data were as follows: alpha fetoprotein (AFP): 704.60 ng/mL (reference range: 0–8 ng/mL), enolase (NSE): 43.29 ng/mL (reference range: 0–3.4 ng/mL), serum carbohydrate antigen (CA)-199: 86.70 U/mL (reference range: 0–22 U/mL), and normal liver and kidney function tests.

An exploratory laparotomy was performed after preparation prior to surgery. During operation, intraoperative findings displayed a huge mass originating from the pancreatic head with high surface tension and a complete capsule. The duodenum and superior mesenteric vessels were involved, making the separation of the lesion from the surrounding involved tissue difficult. A subsequent pylorus-preserving pancreaticoduodenectomy (PPPD) procedure was ultimately performed on the patient. The entire mass was resected from the head of the pancreas and sent to the laboratory for frozen section evaluation.

**Figure 3:**
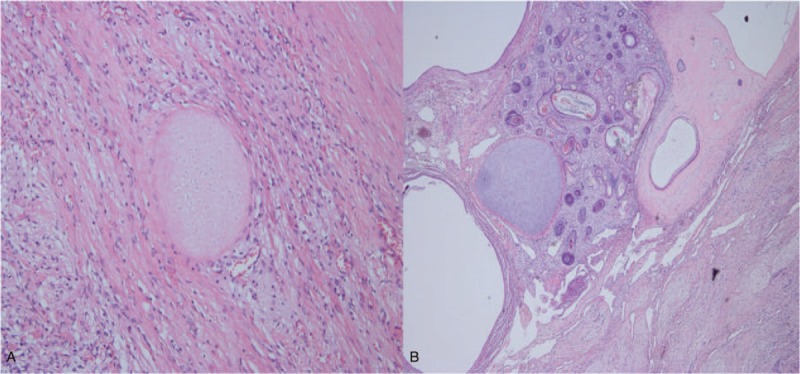
(A, B). Pathological findings of the cystic lesion. A. Hair follicle tissue and cartilage tissue in this pathological section (H&E stain). B. The keratinized squamous epithelium formed the wall of the tumor cyst (H&E stain).

In our case, gross pathological evaluation presented a mass that was 12 cm × 9 cm × 8 cm in size. Histologically, the mass contained abundant keratinous debris and components of adipose tissue, cartilage, and hair follicle. The morphological features were consistent with a mature cystic teratoma of the pancreas, a type of benign tumor (Fig. 3). Teratomas are classified in accordance with the classification of the World Health Organization as mature and immature on the basis of morphological characteristics and differentiation degree.

Postoperatively, the patient developed fever and pulmonary infections that required antibiotic treatment of bacteremia but did not experience PPPD-associated mortalities, such as pancreatic leak, delayed gastric emptying, and fat malabsorption. The boy was discharged on postoperative day 22. At 3 months postoperative, the AFP level decreased to normal range, and the patient did not complain of any discomfort.

## 3 Discussion

Kerr^[3]^ first described pancreatic teratoma in 1918. To date, 9 pediatric patients with pancreatic teratomas have been reported in the English-language literature, as summarized in Table 1. Clinically, pediatric pancreatic teratoma (PPT) presents with nonspecific symptoms, and most of these cases manifest as a palpable abdominal mass and/or tenderness.^[11]^

**Table 1:**
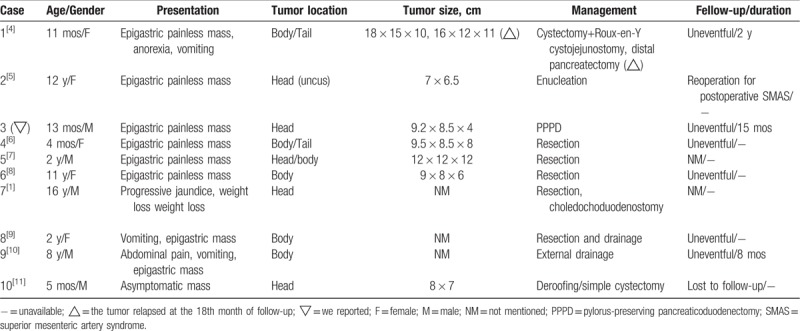
Reported cases on pediatric pancreatic teratomas in the English literature.

The preoperative diagnosis of PPT is difficult. In several cases, the characteristic of fat and calcium combination in the radiologic appearance of magnetic resonance/computerized tomography (MR/CT) is considered highly predictive of mature teratomas. In our case, however, MR and CT only revealed the position and shape of the tumor without this characteristic. Similarly, tumor markers, such as enolase (NSE), carcinoembryonic antigen, and CA-199, lack specificity and sensitivity, except for serum AFP, the level of which is suggestive of disease activity. Endosonography-guided fine needle aspiration biopsy is proposed to help in the preoperative diagnosis of pancreatic tumors; however, this technique is limited by lack of equipment and expertise.^[12]^ Therefore, postoperative pathological evaluation remains the primary diagnostic approach.

With regard to treatment, surgical resection is the mainstay of therapy for PPTs, but detailed surgical guidelines are unavailable. In general, complete surgical resection without damaging vital structures is recommended, while incomplete resection is associated with poor oncological outcome. The operation procedure is determined on the basis of tumor location.^[13]^ PD is a common procedure in the treatment of numerous benign and malignant pancreatic or periampullary diseases. To our knowledge, PD is a rare procedure among the pediatric population because of the low rate of periampullary diseases in this population and the possibility of growth retardation. Notably, PD may lead to impaired exocrine and pancreatic endocrine functions and gastrointestinal problems. Thus, the postoperative endocrine and exocrine functions of the pancreas and the digestive function must be evaluated; moreover, serum AFP level and quality of life must be closely monitored during follow-up.^[14–16]^

In conclusion, PPTs are exceedingly rare tumors. Complete surgical resection combined with follow-up remains the primary treatment. PD is a safe and efficient method for the management of these tumors but must be performed by a surgeon with sophisticated expertise.

## Acknowledgments

The authors gratefully acknowledge the whole staff of the Department of Gastrointestinal Surgery, West China Hospital, who generously provided assistance in the collection of data throughout the duration of the study.

## Author contributions

**Conceptualization:** Yuan Yin, Bo Zhang.

**Data curation:** Jian Wang, Yuan Yin.

**Methodology:** Yuan Yin, Zhaolun Cai, Chaoyong Shen, Xiaonan Yin, Xin Chen, Zhou Zhao.

**Supervision:** Bo Zhang.

**Validation:** Bo Zhang.

**Writing – original draft:** Jian Wang.

**Writing – review & editing:** Bo Zhang.

Bo Zhang orcid: 0000-0002-0254-5843.

Jian Wang orcid: 0000-0001-7388-7368.
